# Vitamin C deficiency after kidney transplantation: a cohort and cross-sectional study of the TransplantLines biobank

**DOI:** 10.1007/s00394-024-03426-7

**Published:** 2024-05-29

**Authors:** Manuela Yepes-Calderón, Yvonne van der Veen, Fernando Martín del Campo S, Daan Kremer, Camilo G. Sotomayor, Tim J. Knobbe, Michel J. Vos, Eva Corpeleijn, Martin H. de Borst, Stephan J. L. Bakker, TransplantLines Investigators

**Affiliations:** 1 Division of Nephrology, Department of Internal Medicine, University Medical Center Groningen, University of Groningen, Hanzeplein 1, Groningen, 9700 RB The Netherlands; 2 Faculty of Medicine, Universidad Nacional Autónoma de México, Mexico City, Mexico; 3 Clinical Hospital University of Chile, Independencia, Santiago, Chile; 4 Institute of Biomedical Sciences, University of Chile, Independencia, Santiago, Chile; 5 Department of Laboratory Medicine, University Medical Center Groningen, University of Groningen, Groningen, The Netherlands; 6 Department of Epidemiology, University Medical Center Groningen, University of Groningen, Groningen, The Netherlands

**Keywords:** Ascorbic acid, Deficiency, Antioxidant

## Abstract

**Purpose:**

Vitamin C deficiency is associated with excess mortality in kidney transplant recipients (KTR). We aim to evaluate plasma vitamin C status at different post-transplantation moments and assess the main characteristics associated with vitamin C deficiency in KTR.

**Methods:**

Plasma vitamin C was assessed in 598 KTR at 3-, 6-, 12-, 24-, and 60-months post-transplantation, 374 late KTR with a functioning graft ≥ 1 year, and 395 potential donors. Vitamin C deficiency was defined as plasma vitamin C ≤ 28 µmol/L. Diet was assessed by a 177-item food frequency questionnaire. Data on vitamin C-containing supplements use were extracted from patient records and verified with the patients.

**Results:**

Vitamin C deficiency ranged from 46% (6-months post-transplantation) to 30% (≥ 1 year post-transplantation). At all time points, KTR had lower plasma vitamin C than potential donors (30–41 µmol/L vs 58 µmol/L). In cross-sectional analyses of the 953 KTR at their first visit ≥ 12 months after transplantation (55 ± 14 years, 62% male, eGFR 55 ± 19 mL/min/1.73 m^2^), the characteristics with the strongest association with vitamin C deficiency were diabetes and smoking (OR 2.67 [95% CI 1.84–3.87] and OR 1.84 [95% CI 1.16–2.91], respectively). Dietary vitamin C intake and vitamin C supplementation were associated with lower odds (OR per 100 mg/day 0.38, 95% CI 0.24–0.61 and OR 0.21, 95% CI 0.09–0.44, respectively).

**Conclusion:**

Vitamin C deficiency is frequent among KTR regardless of the time after transplantation, especially among those with diabetes and active smokers. The prevalence of vitamin C deficiency was lower among KTR with higher vitamin C intake, both dietary and supplemented. Further research is warranted to assess whether correcting this modifiable risk factor could improve survival in KTR.

**Supplementary Information:**

The online version contains supplementary material available at 10.1007/s00394-024-03426-7.

## Introduction

The first line treatment for patients with kidney failure is kidney transplantation, since it offers better survival rates compared to chronic dialysis [1]. Nonetheless, the life expectancy of kidney transplant recipients (KTR) remains shorter compared to age- and sex-matched controls of the general population [2]. Besides the traditional risk factors for mortality, KTR also have an enhanced pro-oxidative and pro-inflammatory status compared to the general population [3, 4], which further contributes to their increased mortality risk [5]. Therefore, there is an ongoing search for potential tools that could help moderate the deleterious effects of these risk factors.

Vitamin C (ascorbic acid) is an essential nutrient in humans and a powerful biological antioxidant which serves as cofactor for multiple human enzymes involved in anti-inflammatory response [6–8]. For its important function in regulating oxidative status, vitamin C has gained attention in the kidney post-transplantation setting, especially because previous evidence has connected vitamin C deficiency in KTR with a two-fold increased risk of all-cause mortality and reduced long-term graft survival [9–11]. It has been proposed that KTR are prone to vitamin C deficiency as a remmant effect of dialysis treatment and dietary potassium restrictions before transplantation, which also restricts fruit and vegetable intake [12, 13]. Furthermore, it has been postulated that vitamin C expenditure could be higher in KTR due to the heightened oxidative and inflammatory status of this population [11, 13]. Nevertheless, the actual prevalence of vitamin C deficiency post-kidney transplantation remains undisclosed. Since vitamin C deficiency is potentially correctable [14, 15], understanding the burden of vitamin C deficiency after kidney transplantation and identifying patients at higher risk is of importance for healthcare providers and future researchers, to plan potential interventions aimed to decrease the burden of premature mortality in KTR.

We hypothesized that vitamin C deficiency is frequent among KTR regardless of their time after transplantation and that KTR at the highest risk for deficiency would be those with prooxidant/proinflammatory insults such as smoking and with a lower vitamin C intake. In the current study, we aim to evaluate plasma vitamin C concentration and status at different moments in the post-kidney transplantation setting and to assess the main clinical and dietary characteristics associated with vitamin C deficiency in KTR.

## Material and methods

### Study design

In the TransplantLines Biobank and Cohort Study (Clinical trials identifier: NCT03272841) [16], all KTR who visited the outpatient clinic of the University Medical Centre of Groningen (The Netherlands) were included by convenience sample into two non-overlapping cohorts: (i) in cohort A, 819 patients were included prior to their kidney transplantation and attended study visits at 3-, 6-, 12-, 24- and 60-months post-transplantation; (ii) in cohort B, 688 KTR were included who were already at ≥ 12 months post-transplantation at invitation for participation in the cohort study and attended a single study visit at inclusion. All KTR were, in principle, eligible unless they fulfilled exclusion criteria, which were age < 18 years old, no mastery of the Dutch language, or no capability to intellectually comprehend questionnaires or physical tests. Vitamin C measurements were included as part of the standard study visit protocol between December 2016 and March 2022, resulting in 598 KTR from cohort A and 374 from cohort B with at least one plasma vitamin C measurement available, which is the population of the current study. A detailed Consort flowchart is presented in Supplemental Fig. 1. Additionally, 395 potential donors were recruited by convenience sample during their pre-transplantation assessment, were invited to participate, and provided plasma samples to serve as healthy controls. All patients provided written informed consent. The TransplantLines Biobank and Cohort Study was approved by the UMCG institutional review board (METc 2014/077), adheres to the UMCG Biobank Regulation, and is in accordance with the Declarations of Helsinki and Istanbul.

**Fig. 1:**
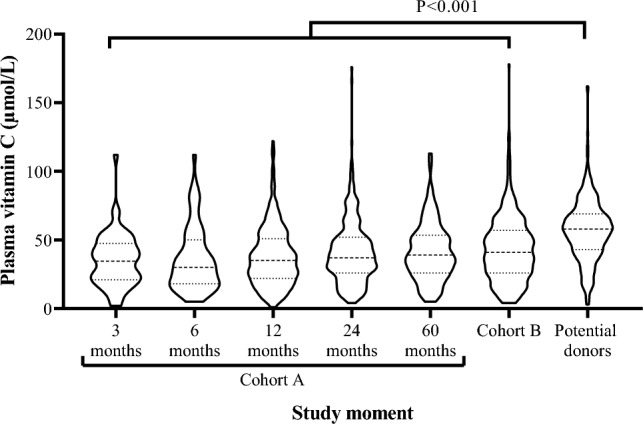
Vitamin C concentration in KTR. Dashed and dotted lines represent the median and first and third quartile, respectively, and the width of the sections represent the probability that members of the population will take that given value. The median (IQR) plasma vitamin C concentration was 35 (21–48), 30 (18–50), 35 (22–51), 37 (26–52), 39 (26–54), 41 (26–57), and 58 (43–69) µmol/L at 3, 6-, 12-, 24- and 60-month post-transplantation, among prevalent KTR of cohort B and among potential donors, respectively

### Data collection

During every study visit, patients were assessed by a physician or a medical student who registered weight and height while participants wore indoor clothing without shoes; the body mass index was later calculated. Blood pressure was also measured as the average of three measurements with 1-min intervals after a 6-min rest in a supine position using an automatic device (Omron M4, Omron Europe B.V., Hoofddorp, The Netherlands).

Dietary intake was recorded through a semi-quantitative self-administered food frequency questionnaire (FFQ) of 177 items at 12 months post-transplantation for cohort A and at the first study visit for cohort B. The questionnaires were self-fulfilled by patients before the study visit and later verified by study personnel. This FFQ was developed at Wageningen University and has been previously validated for the Dutch population [17]. It records dietary intake information during the past month in natural units (e.g., a slice of bread) or household measures (e.g., a teaspoon). Data were then converted into total energy and food group intake per day using the 2006 Dutch Food Composition Table [18]. Data on use of vitamin C containing supplements were extracted from the electronic patient record and verified via telephone with the patients. Vitamin C supplementation was recorded as positive (patient confirmed supplementation) or negative (patient denied or was unsure). Patients did not receive any instructions about supplement use.

Finally, other relevant clinical information was extracted from the electronic patient records. Stable variables were extracted at inclusion, including demographics (Date of birth and sex) and transplantation characteristics (date of transplantation, Pre-emptive transplantation, Dialysis duration). Dynamic variables were extracted at each study visit, including current comorbidities (history of cardiovascular disease [defined as ICD-9 codes 410–447] and active smoking), medication for chronic diseases (use of statins, antihypertensives, proton pump inhibitors, and iron supplementation) and immunosuppression (use of Prednisolone, Cyclosporine, Tacrolimus, Mycophenolic acid, and Everolimus) [16].

### Biochemical analyses, calculations, and definitions

Before every study visit, all KTR were asked to collect a 24-h urine sample the day before. On the day of the study visit, they also supplied a blood sample in the morning after a minimum of 8 to 12 h of overnight fasting period, including no medication use.

For the assessment of vitamin C concentration, directly after blood sampling, the blood was cooled, deproteinized, and stored at -20 ºC. Next, vitamin C was transformed to dehydroascorbic acid and later derivatized to 3-(1,2-hydroxyethyl) furo-[3,4-b] quinoxaline-1-one. Finally, vitamin C plasma concentration was obtained by reversed-phase liquid chromatography with fluorescence detections (excitation 355 nm, emission 425 nm) [19]. Vitamin C deficiency was defined as a plasma vitamin C concentration ≤ 28 µmol/L [11].

For the remaining laboratory measurements, after the conclusion of the study visit, blood and urine samples were sent to the hospital laboratory. Clinical chemistry assays were then conducted encompassing parameters related to kidney function (creatinine, urinary protein excretion, and urinary potassium excretion), glucose homeostasis (glycated hemoglobin), lipid profile (total cholesterol and HDL cholesterol), and inflammation (hs-CRP and albumin). These assays were performed using routine spectrophotometric methods in clinical practice (Roche Diagnostics, Basel, Switzerland). The estimated glomerular filtration rate (eGFR) was calculated using the 2021 creatinine-based Chronic Kidney Disease Epidemiology Collaboration formula [20]. Diabetes was defined as HbA_1C_ ≥ 48 mmol/mol, non-fasting glucose ≥ 11.0 mmol/l, fasting glucose ≥ 7.0 mmol/l, or the use of diabetes medication according to the criteria of the American Diabetes Association [21].

### Statistical analyses

We performed data analyses, computations, and created graphs with GraphPad Prism version 7 software (GraphPad Software, San Diego, CA) and R version 4.0.5 (R Foundation for Statistical Computing, Indiana, USA). Data are presented as mean ± standard deviation (SD) for normally distributed data and as median (interquartile range [IQR]) for variables with a non-normal distribution. Quantile–quantile plots were used to assess normality. Categorical data are expressed as numbers (valid percentages).

For all follow-up moments, we calculated the median (IQR) plasma vitamin C concentration and the proportion of KTR who were vitamin C deficient. We then compared the median plasma vitamin C concentration and the proportion of vitamin C deficiency to that of potential donors using Kruskal Wallis test with Dunn’s multiple comparisons test and χ^2^ test. Among the individuals with repeated measurements, we also explored the change of plasma vitamin C concentrations over time. In order to take into account the potential intra-individual variation, the group trajectory was calculated by fitting an unconditional growth model using time after transplantation as the time variable. Individual trajectories were also graphed in a scatter plot.

Differences at baseline among subgroups of patients were tested by one-way ANOVA for continuous variables with normal distribution, Mann–Whitney U test for continuous variables with non-normal distribution, and χ^2^ test for categorical variables. A statistical significance level of P < 0.05 (two-tailed) was used for all other analyses.

### Vitamin C deficiency determinants

For the cross-sectional analyses, cohort A and cohort B were combined, taking for cohort A, the data from the first visit at ≥ 12 months post-transplantation, resulting in a group of 953 KTR (Supplemental Fig. 1). Logistic regression analyses with backward selection (P_in_ = 0.05, P_out_ = 0.10) were performed to select the clinical characteristics more significantly associated with vitamin C deficiency. Then, we used logistic regression analysis with vitamin C deficiency as the outcome to explore whether it was associated with food group intake, vitamin C intake, and vitamin C supplementation as the independent variables. These analyses were performed crude and adjusted for the variables chosen through the prior backward selection analyses. Models were checked for the fulfillment of logistic regression assumptions.

### Secondary analyses

As secondary analyses, we used linear regression analysis with vitamin C plasma concentration as the outcome to explore whether it was associated with food group intake, vitamin C intake, and vitamin C supplementation as the independent variables. These analyses were performed crude and adjusted for the variables chosen through the prior backward selection analyses. Models were checked for the fulfillment of linear regression assumptions. We also evaluated whether age, sex, or the variables previously identified in the backward selection analyses had additive interaction concerning the association between vitamin C intake/supplementation with vitamin C deficiency as outcome variable. To do so, we introduced a multiplicative term between the variable of interest and Vitamin C intake/supplementation in the logistic regression analyses. These analyses were permformed crude and with adjustment for the other variables in the clinical model plus age and sex.

## Results

### Patient characteristics

The patient characteristics of KTR from cohorts A and B at their first visit ≥ 12 months post-transplantation are presented in Table 1. In this study population, the median plasma vitamin C concentration was 34 (24–53) µmol/L, mean age was 55 ± 14 years, 62% were male, and mean eGFR was 55 ± 19 mL/min/1.73 m^2^. For cohort B, the median time after transplantation was 8 (4–14) years.

**Table 1:** Patient characteristics of the study population

Patient characteristics	Overall KTR	Plasma vitamin C concentration		P value
		≤ 28 µmol/L	> 28 µmol/L	
*n*	953	322	631	
Vitamin C, µmol/L	37 (24–53)	18 (13–24)	47 (37–60)	< 0.001
Demographics				
Age, years	55 ± 14	57 ± 13	55 ± 14	0.007
Sex (male), *n* (%)	590 (62)	226 (70)	364 (58)	< 0.001
Body mass index, kg/m^2a^	27.5 ± 4.7	28.3 ± 4.8	27.0 ± 4.6	< 0.001
Graft function				
eGFR, mL/min/1.73 m^2^	55 ± 19	52 ± 20	57 ± 18	< 0.001
Urinary protein excretion, g/24-h^b^	0.2 (0.1–0.3)	0.2 (0.1–0.4)	0.2 (0.1–0.3)	0.02
Urinary potassium excretion, mmol/24-h	71.9 ± 24.4	67.8 ± 24.5	73.7 ± 24.2	0.02
Transplant characteristics				
Pre-emptive transplantation, *n* (%)	304 (32)	92 (29)	212 (34)	0.13
Dialysis duration, months^c^	9 (0–28)	14 (0–30)	6 (0–25)	0.01
Time after transplantation, years^d^	1 (1–6)	1 (1–4)	1 (1–7)	0.01
Cardiovascular history				
Systolic blood pressure, mmHg^e^	134 ± 16	135 ± 16.6	134 ± 14.9	0.54
Active smoking (yes), *n* (%)^f^	138 (15)	65 (20)	73 (12)	0.001
Diabetes, *n* (%)^g^	264 (28)	133 (42)	131 (21)	< 0.001
Statine use, *n* (%)	548 (58)	199 (62)	349 (55)	0.07
Antihypertensive use, *n* (%)	803 (84)	284 (88)	519 (82)	0.02
Laboratory				
Glycated hemoglobin, %^h^	5.8 (5.4–6.4)	6.0 (5.6–6.8)	5.7 (5.4–6.2)	< 0.001
hs-CRP, mg/l^i^	1.9 (0.8–4.7)	2.5 (1.0–6.0)	1.7 (0.6–4.2)	< 0.001
Albumin, g/l^j^	43.6 ± 2.9	43.2 ± 3.0	43.8 ± 2.9	0.002
Total cholesterol, mmol/l^h^	4.61 ± 0.99	4.51 ± 1.03	4.65 ± 0.97	0.04
HDL cholesterol, mmol/l^j^	1.38 ± 0.42	1.27 ± 0.38	1.44 ± 0.42	< 0.001
Immunosuppression				
Prednisolone use, *n* (%)	928 (98)	314 (98)	614 (97)	1.00
Cyclosporine use, *n* (%)	104 (11)	23 (7)	81 (13)	0.01
Tacrolimus use, *n* (%)	736 (77)	274 (85)	462 (73)	< 0.001
Mycophenolic acid use, *n* (%)^k^	770 (81)	264 (82)	506 (80)	0.50
Everolimus use, *n* (%)	44 (5)	16 (5)	28 (4)	0.84
Azathioprine use, *n* (%)	63 (7)	16 (5)	47 (7)	0.19
Other medication				
Proton pump inhibitor use, *n* (%)	684 (72)	250 (78)	434 (69)	0.01
Iron supplementation, *n* (%)	70 (74)	24 (7)	46 (7)	1.00
Diet				
Fruit, g/d^l^	163 ± 123	137 ± 110	175 ± 127	0.001
Vegetable, g/d^l^	115 ± 79	112 ± 84	116 ± 76	0.69
Dairy, g/d^l^	349 ± 239	362 ± 247	343 ± 236	0.41
Meat, g/d^l^	117 ± 74	111 ± 68	119 ± 76	0.20
Fish, g/d^l^	24. ± 34	20 ± 29	26 ± 35	0.07
Nuts, g/d^l^	5 (0–18)	4.6 (0–16)	5 (0–19)	0.20
Bread, g/d^l^	156 ± 90.7	156 ± 90.7	156 ± 90.9	0.98
Coffee, mL/d^l^	420 (240–561)	420 (200–561)	420 (240–561)	0.83
Tea, mL/d^l^	291 (30–510)	243 (8–510)	291 (79–510)	0.08
Dietary vitamin C intake, mg/d^m^	97 ± 48	84 ± 44	103 ± 48	< 0.001
Vitamin C supplementation, *n* (%)^n^	69 (10)	7 (3)	62 (13)	< 0.001

^¥^Differences were tested by ANOVA for continuous variables with normal distribution, Kruskal–Wallis test for continuous variables with non‒normal distribution, and by *χ*^2^ test for categorical variables

Data available in ^a^939, ^b^848, ^c^779, ^d^911, ^e^936, ^f^942, ^g^928, ^h^949, ^i^920, ^j^950, ^k^952, ^l^500, ^m^487 and ^n^688 KTR

### Vitamin C after transplantation

The median (IQR) plasma vitamin C concentration was 35, 30, 35, 37, and 39 µmol/L at 3-, 6-, 12-, 24-, and 60-month post-transplantation, respectively, in cohort A and 41 µmol/L among the KTR from cohort B. Potential donors had a median vitamin C concentration of 58 µmol/L. At all time points, the plasma vitamin C concentration of KTR was significantly lower than that of potential donors (Fig. 1).

**Fig. 2:**
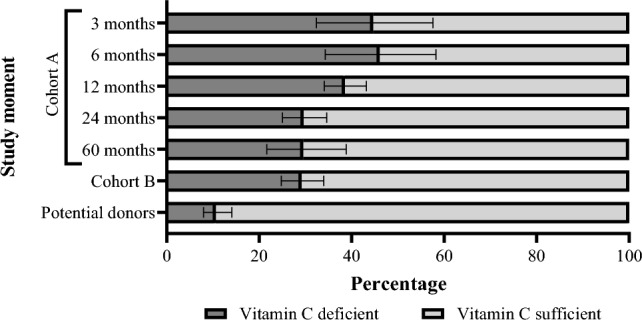
Vitamin C status in KTR. The percentage of vitamin C deficient KTR was 45, 46, 39, 30, 30, 30 and 11% at 3-, 6-, 12-, 24- and 60-month post-transplantation, among prevalent KTR of cohort B and among potential donors, respectively. P < 0.001 for χ^2^ test

The prevalence of vitamin C deficiency among KTR was 45, 46, 39, 30, and 30% at 3-, 6-, 12-, 24, and 60-month post-transplantation, respectively, and 30% among the KTR from cohort B. The prevalence of vitamin C deficiency in potential donors was 11%. At all time points, the prevalence of vitamin C deficiency in KTR was higher than that of potential donors (Fig. 2).

Fitting an unconditional growth model revealed that, on average, vitamin C increased by 1.57 µmol/L per year after transplantation (Supplemental Fig. 2).

### Vitamin C deficient KTR

At the cross-sectional analyses at ≥ 12 months post-transplantation, vitamin C-deficient KTR, in comparison to those vitamin C sufficient, were older, more frequently male, and had higher BMI. Their graft function appeared to be worse with a lower eGFR and a higher urinary protein excretion. They were also more recently transplanted. Among vitamin C deficient and sufficient KTR, there were no differences regarding their dialysis time before transplantation or the proportion of patients with pre-emptive transplantation. Next, vitamin C-deficient KTR seemed to have a less favorable cardiovascular profile, with a higher prevalence of active smoking, more frequent antihypertensive use, higher glycated hemoglobin, higher HDL cholesterol, and higher hs-CRP. Regarding medication, vitamin C deficient KTR more frequently used tacrolimus as part of immunosuppressive therapy and more frequently used proton pump inhibitors. Finally, when evaluating diet, on average, KTR in both groups ate an amount of vitamin C between the recommended daily amount (RDA) of 75 and 110 mg/day. However, among vitamin C deficient KTR, the average vitamin C intake was 19 mg/day lower than of vitamin C sufficient KTR, 33% did not fulfill the vitamin C RDA, and their fruit intake was also lower. Their urinary potassium excretion was also lower, indicating lower potassium intake. Vitamin C supplementation was also less frequently used among these KTR (Table 1).

### Independent determinants of vitamin C deficiency

In logistic regression analyses with backward selection, the most important independent clinical characteristics associated with higher odds of vitamin C deficiency were diabetes (OR 2.67) and active smoking (OR = 1.84). The variables inversely associated with the odds of vitamin C deficiency were HDL cholesterol (OR per SD increase = 0.67), eGFR (OR per SD increase = 0.76), plasma albumin (OR per SD increase = 0.83), and time since transplantation (OR per year = 0.94, Table 2).

**Table 2:** Clinical determinants of vitamin C deficiency

Baseline characteristics	Vitamin C deficiency		
	Odds ratio	95% CI	P value
Diabetes, yes	2.67	1.84–3.87	< 0.001
Active smoking, yes	1.84	1.16–2.91	0.01
HDL cholesterol, per SD increase	0.67	0.55–0.82	< 0.001
eGFR, per SD increase	0.76	0.63–0.91	0.003
Plasma albumin, per SD increase	0.83	0.69–0.99	0.04
Time since transplantation, years	0.94	0.90–0.99	0.02

Logistic regression analyses with backward selection were performed (P_in_ 0.05, P_out_ 0.10). Age, sex, body mass index, estimated glomerular filtration rate, urinary protein excretion, pre-emptive transplantation, dialysis duration, systolic blood pressure, statin use, antihypertensive use, glycated hemoglobin, hs-CRP, total cholesterol, prednisolone use, cyclosporine use, tacrolimus use, mycophenolic acid use, azathioprine use, proton pump inhibitor use, and iron supplementation use were eliminated from the final model

When assessing the diet of KTR, the food groups associated with a minor risk of vitamin C deficiency were fruit (OR per 100 g = 0.75) and nuts (OR per 100 g = 0.30). Additionally, higher vitamin C intake from the diet (OR per 100 g = 0.38) and from supplementation (OR = 0.21) were associated with a reduced risk of vitamin C deficiency. After adjustment for the clinical variables selected in the previous model, nut intake was no longer significantly associated with vitamin C deficiency (Table 3).

**Table 3:** Dietary determinants of plasma vitamin C deficiency in KTR

	Vitamin C deficiency					
	Crude			Adjusted^†^		
	Odds Ratio	95% CI	P value	Odds Ratio	95% CI	P value
Fruit, 100 g/d	0.75	0.62–0.89	0.002	0.78	0.64–0.94	0.01
Vegetable, 100 g/d	0.95	0.74–1.21	0.68	0.97	0.74–1.27	0.84
Dairy, 100 g/d	1.03	0.96–1.12	0.40	1.01	0.92–1.10	0.88
Meat, 100 g/d	0.85	0.65–1.10	0.22	0.84	0.62–1.12	0.24
Fish, 100 g/d	0.58	0.30–1.05	0.09	0.62	0.31–1.19	0.17
Nuts, 100 g/d	0.30	0.09–0.86	0.03	0.52	0.16–1.62	0.28
Bread, 100 g/d	1.00	0.81–1.23	0.98	1.01	0.79–1.28	0.95
Coffee, 100 ml/d	0.99	0.93–1.07	0.97	0.99	0.92–1.08	0.90
Tea, 100 ml/d	0.98	0.93–1.03	0.42	1.00	0.94–1.06	0.96
Vitamin C intake, 100 mg/d	0.38	0.24–0.61	< 0.001	0.44	0.26–0.72	0.002
Vitamin C supplementation use, yes	0.21	0.09–0.44	< 0.001	0.25	0.10–0.55	0.001

Univariable and multivariable logistic regression analyses were performed

^**†**^Adjustment was performed in each logistic regression analysis by history of diabetes, active smoking status, HDL cholesterol, estimated glomerular filtration rate, plasma albumin concentration, and time since transplantation

### Secondary analyses

In secondary linear regression analyses, fruit intake was most strongly positively associated with higher plasma vitamin C concentration. Furthermore, there was a positive association of fish and nuts intake with plasma vitamin C concentration. Consistently, dietary vitamin C intake and intake with supplementation were also positively associated with higher plasma vitamin C concentration. After adjustment for the clinical variables selected in the backward model, nut intake was no longer significantly associated with plasma vitamin C (Supplemental Table 1). KTR, with higher fish intake, also had a higher fruit, vegetable, and nut intake and lower bread intake (Supplemental Table 2).

In effect-modification analyses, neither of the clinical variables selected in the backward model nor age and sex were significant effect modifiers of the association of vitamin C intake and supplementation with vitamin C deficiency risk (Supplemental Table 3).

## Discussion

In two large cohorts of outpatient KTR, we aimed to evaluate plasma vitamin C status at different post-transplantation moments and assess the main characteristics associated with vitamin C deficiency. We showed that vitamin C deficiency was highly prevalent in this population, peaking at six months post-transplantation at 46% and later stabilizing at around 30% from 24 months post-transplantation onwards. At any moment post-transplantation, KTR were at least three times more likely to be vitamin C deficient than potential donors, and their median plasma vitamin C concentration was also always lower. The main clinical characteristics associated with a higher risk of vitamin C deficiency in KTR were diabetes and active smoking. Additionally, we found that higher vitamin C intake, both from diet, particularly fruit intake, and from supplementation, were associated with a lower risk of vitamin C deficiency. These results describe for the first time the continuous burden of vitamin C deficiency among KTR and are of importance to future researchers and the healthcare community to consider vitamin C deficiency as a frequently occurring, potentially modifiable risk factor for KTR survival. They also provide healthcare professionals with tools to identify patients who could benefit from (dietary) interventions to improve their vitamin C status.

Multiple mechanisms have been proposed as a potential explanation for the high rate of vitamin C deficiency among KTR, including (i) a potential remanent effect of long-term dialysis [12, 13], (ii) a lower intake of vitamin C-rich food if KTR do not adapt the recommendations they had before kidney transplantation for the purpose of restricting potassium intake [13], and (iii) higher vitamin C requirements do to their enhanced oxidative and inflammatory status [9], especially present in the early post-transplantation, but chronically withstanding [3, 5]. Regarding mechanism (i), we did not find an association between dialysis vintage or pre-emptive transplantation and vitamin C deficiency, suggesting that the effects of dialysis on vitamin C status are not of particular impact after transplantation. Regarding mechanism (ii), we found that our study population at ≥ 12 months post-transplantation consumed, on average, an amount of vitamin C between the recommended daily amount (RDA) of 75 and 110 mg/day [8], consistent with the current clinical guidelines that do not consider dietary restrictions for vitamin C or vitamin C rich-food among KTR [22]. However, among vitamin C-deficient KTR, 33% did not fulfill the vitamin C RDA, suggesting that low intake is also a contributing factor to vitamin C deficiency for these patients. Finally, regarding the mechanism (iii), given that KTR had an average intake according to the RDA but still had high prevalence of vitamin C deficiency, is reasonable to thik that their requirements could also be higher. We also found that vitamin C-deficient KTR had higher concentrations of biomarkers of acute-phase inflammation, such as hs-CRP. This could suggest either an enhanced inflammatory status in vitamin C deficient KTR or more vitamin C expenditure in KTR with enhanced acute inflammation. The cross-sectional nature of our study does not allow us to distinguish the directionality of the association.

In the current study, we found multiple clinical characteristics that associated a with higher odds of vitamin C deficiency, such as diabetes. This finding is consistent with evidence in the chronic kidney disease setting that found that patients with chronic kidney disease who had diabetes, on average, had a 10 µmol/L lower concentration of plasma vitamin C than patients without diabetes [23]. One potential explanation is that the dietary recommendations of decreasing sugar intake in patients with diabetes among the KTR population decrease their fruit intake and, therefore, their vitamin C intake [24]. Another explanation it is known that patients with diabetes have an enhanced pro-inflammatory and pro-oxidant status [25], which could increase their vitamin C expenditure. A similar mechanism has been proposed for smoking [26], the other clinical characteristic that we found to be associated with higher odds of vitamin C deficiency. Previous studies on the relationship between vitamin C intake and status in the general population had already proposed that smokers require vitamin C intakes ~ twofold higher than non-smokers to reach adequate vitamin C concentrations[26].

We also found clinical characteristics that are associated with lower odds of vitamin C deficiency, such as eGFR. A previous study had already reported an association between impaired kidney function and lower plasma vitamin C concentration, which, according to the authors, related to damage to the tubular reabsorption mechanisms for vitamin C and, therefore, increased urinary loss [23]. Next, we found an inverse association between time since transplantation and vitamin C deficiency. Although vitamin C deficiency was frequent at all moments post-transplantation, it appeared that the odds were especially higher in the first year of transplantation. This could be related to (i) the recovery of pre-existing deficiency present during kidney failure. (ii) a stabilization period that occurs during the first year of transplantation regarding losing the habit of eating with the dietary restrictions common to the period of kidney failure prior to transplantation [27]. (iii) the introduction and titration of immunuspresive therapy, many of which have gastrointestinal effects that can affect appetite [28]. And (iv) the consequences of the transplantation procedure itself, which generates ischemia–reperfusion injury [29], increasing the oxidative stress load and, potentially, vitamin C requirements in the first months after transplantation. Finally, we showed that nutrition plays a vital role in vitamin C status among KTR. First, we found an inverse association between plasma albumin concentration and the odds of vitamin C deficiency, suggesting that KTR, with better general nutritional status, also were less prone to vitamin C deficiency. Furthermore, we showed that a higher dietary intake of vitamin C and vitamin C-rich food groups did reflect in an average lower odds of vitamin C deficiency and a higher plasma vitamin C concentration. This was also the case for patients with dietary patterns characterized by a higher intake of fish, nuts, and vegetables. Vitamin C supplementation was also significantly associated with an almost 80% decreased relative risk of vitamin C deficiency.

Among KTR, vitamin C deficiency has been associated with a two-fold increase in the risk of cancer-related and all-cause mortality in KTR [10, 11]. Vitamin C deficiency has also been described as an independent risk factor for reduced long-term graft survival [9]. This evidence has brought attention to potential possibilities to improve vitamin C status in this population. One option would be vitamin C supplementation. In the general population, vitamin C supplementation between 90 mg to 3 gr per day is considered relatively safe, with mild adverse effects such as gastrointestinal disturbances and fatigue [13]. Although evidence is less available in the specific post-transplantation setting, few interventional studies have also reported little adverse effects and have shown improvements in antioxidant capacity and eGFR [13, 30]. However, Vitamin C is naturally present in sufficient quantities in various foods, especially fruits and vegetables [31]. Since we also found an association with the dietary intake of vitamin C, we consider that optimizing the intake of vitamin C-rich foods should be the first considered potential therapeutic approach in KTR to reduce the burden of vitamin C deficiency, especially because vitamin C-rich eating patterns have already been associated with a lower mortality risk in KTR [32]. More evidence is needed to conclude whether vitamin C supplementation could also be of benefit in this population.

These results describe for the first time the continuous burden of vitamin C deficiency among KTR and are of importance to future researchers and the healthcare community to consider vitamin C deficiency as a frequently occurring, potentially modifiable risk factor for KTR survival. They also provide healthcare professionals with tools to identify patients who could benefit from (dietary) interventions to improve their vitamin C status.

The present study has several strengths. To the extent of our knowledge, it comprises the largest KTR cohort in which plasma vitamin C has been assessed, and furthermore, we counted with repeated measurements that allowed us to assess vitamin C trajectory. Also, our extensively phenotyped cohort allowed us to evaluate the association of vitamin C deficiency with multiple clinical variables and detailed dietary information. Finally, we measured vitamin C by evaluating both ascorbic acid and dehydroascorbic acid, the most reliable measure of vitamin C pool [7, 33]. The present study also has several limitations. The original study protocol did not contemplate multiple FFQ post-transplantation, and therefore, we can not describe changes in vitamin C intake. However, we do not expect significant changes in vitamin C intake because KTR are not subject to specific dietary recommendations or interventions regarding vitamin C intake or vitamin C-rich food intake in common clinical practice [22]. Next, the observational and cross-sectional nature of the study does not allow us to conclude the directionality of the associations we found with vitamin C deficiency. In addition, our study was also carried out in a center with a predominantly Caucasian population, which calls for prudence in extrapolating our results to people of other ethnicities and with different dietary patterns. Furthermore, we did not have a biochemical measurement of oxidative stress which would have allowed us to support the mechanistic role of vitamin C as antioxidant defense. Finally, it could be argued that potential donors might not be a fair comparison since they might represent an ultra-healthy population. However, the average plasma vitamin C concentration we found in potential donors was consistent with previous reports obtained from the general population [26, 34]. Further studies would benefit from registering vitamin C intake at multiple time points and considering evaluating oxidative stress biomarkers to support a mechanistic effect of vitamin C.

In conclusion, vitamin C deficiency is frequent among KTR regardless of their time after transplantation. Patient characteristics associated with a higher prevalence of vitamin C deficiency included the presence of prooxidative/proinflammatory insults such as diabetes and smoking. Higher vitamin C intake, both dietary and supplemented, was associated with a lower prevalence of vitamin C deficiency.

## Supplementary Information

Below is the link to the electronic supplementary material.

Supplementary file1 (DOCX 86 KB)

## Data Availability

The data that support the findings of this study are not publicly available due to privacy of research participants but are available from principal investigator S.J.L.B (s.j.l.bakker@umcg.nl) upon reasonable request.

## References

[1] Hariharan S, Israni AK, Danovitch G (2021) Long-term survival after kidney transplantation. N Engl J Med 385:729–743. 10.1056/NEJMra2014530 34407344 10.1056/NEJMra2014530

[2] Vinson AJ, Singh S, Chadban S et al (2022) Premature death in kidney transplant recipients: the time for trials is now. J Am Soc Nephrol 33:665–673. 10.1681/ASN.2021111517 35292438 10.1681/ASN.2021111517 PMC8970447

[3] Fonseca I, Reguengo H, Almeida M et al (2014) Oxidative stress in kidney transplantation. Transplantation 97:1058–1065. 10.1097/01.TP.0000438626.91095.50 24406454 10.1097/01.TP.0000438626.91095.50

[4] Heldal TF, Åsberg A, Ueland T et al (2022) Inflammation in the early phase after kidney transplantation is associated with increased long-term all-cause mortality. Am J Transplant 22:2016–2027. 10.1111/ajt.17047 35352462 10.1111/ajt.17047 PMC9540645

[5] Yepes-Calderón M, Sotomayor CG, Gans ROB et al (2020) Post-transplantation plasma malondialdehyde is associated with cardiovascular mortality in renal transplant recipients: A prospective cohort study. Nephrol Dial Transplant 35:512–519. 10.1093/ndt/gfz288 32133530 10.1093/ndt/gfz288 PMC7056950

[6] Padayatty SJ, Levine M (2016) Vitamin C: the known and the unknown and Goldilocks. Oral Dis 22:463–493 26808119 10.1111/odi.12446 PMC4959991

[7] Granger M, Eck P (2018) Dietary Vitamin C in Human Health. In: Advances in Food and Nutrition Research. Academic Press Inc., pp 281–310 10.1016/bs.afnr.2017.11.006 29477224

[8] National Institute of Health (2021) Vitamin C, Fact Sheet for Health Professionals. In: Dietary Supplemental Fact Sheets. https://ods.od.nih.gov/factsheets/VitaminC-HealthProfessional/. Accessed 6 Mar 2024

[9] Sotomayor CG, Bustos NI, Yepes-Calderon M et al (2021) Plasma vitamin C and risk of late graft failure in kidney transplant recipients: results of the transplantlines biobank and cohort study. Antioxidants 10:631. 10.3390/antiox10050631 33919075 10.3390/antiox10050631 PMC8143099

[10] Gacitúa TA, Sotomayor CG, Groothof D et al (2019) Plasma vitamin C and cancer mortality in kidney transplant recipients. J Clin Med 8:2064. 10.3390/jcm8122064 31771233 10.3390/jcm8122064 PMC6947225

[11] Sotomayor CG, Eisenga MF, Gomes Neto AW et al (2017) Vitamin C depletion and all-cause mortality in renal transplant recipients. Nutrients 9:568. 10.3390/nu9060568 28574431 10.3390/nu9060568 PMC5490547

[12] Sirover WD, Liu Y, Logan A et al (2015) Plasma ascorbic acid concentrations in prevalent patients with end-stage renal disease on hemodialysis. J Ren Nutr 25:292–300. 10.1053/j.jrn.2014.09.007 25455040 10.1053/j.jrn.2014.09.007

[13] Borran M, Dashti-Khavidaki S, Alamdari A, Naderi N (2021) Vitamin C and kidney transplantation: Nutritional status, potential efficacy, safety, and interactions. Clin Nutr ESPEN 41:1–9. 10.1016/j.clnesp.2020.12.017 33487249 10.1016/j.clnesp.2020.12.017

[14] Brubacher D, Moser U, Jordan P (2000) Vitamin C concentrations in plasma as a function of intake: a meta-analysis. Int J Vitam Nutr Res 70:226–237. 10.1024/0300-9831.70.5.226 11068703 10.1024/0300-9831.70.5.226

[15] Choi J, Kim D-Y, Choue R, Lim H (2017) Effects of Vitamin C supplementation on plasma and urinary vitamin c concentration in korean women. Clin Nutr Res 6:198. 10.7762/cnr.2017.6.3.198 28770182 10.7762/cnr.2017.6.3.198 PMC5539213

[16] Eisenga MF, Gomes-Neto AW, van Londen M et al (2018) Rationale and design of TransplantLines: a prospective cohort study and biobank of solid organ transplant recipients. BMJ Open 8:e024502. 10.1136/bmjopen-2018-024502 30598488 10.1136/bmjopen-2018-024502 PMC6318532

[17] Feunekes GIJ, Van Staveren WA, De Vries JHM et al (1993) Relative and biomarker-based validity of a food-frequency questionnaire estimating intake of fats and cholesterol. Am J Clin Nutr 58:489–496. 10.1093/ajcn/58.4.489 8379504 10.1093/ajcn/58.4.489

[18] Stichting Nederlands Voedingsstoffenbestand (2006) NEVO-tabel: Nederlands Voedingsstoffenbestand 2006. Den Haag: Voedingscentrum

[19] Speek AJ, Schrijver J, Schreurs WHP (1984) Fluorometric determination of total vitamin C in whole blood by high-performance liquid chromatography with pre-column derivatization. J Chromatogr B Biomed Sci Appl 305:53–60. 10.1016/S0378-4347(00)83313-7 10.1016/S0378-4347(00)83313-7 6707154

[20] Inker LA, Eneanya ND, Coresh J et al (2021) New creatinine- and cystatin C-based equations to estimate GFR without race. N Engl J Med 385:1737–1749. 10.1056/NEJMoa2102953 34554658 10.1056/NEJMoa2102953 PMC8822996

[21] American Diabetes Association (2014) Diagnosis and classification of diabetes mellitus. Diabetes Care 37:S81–S90. 10.2337/dc14-S081 24357215 10.2337/dc14-S081

[22] Josephson MA (2013) The KDIGO clinical practice guideline for the care of kidney transplant recipients. Kidney News 5:21–22

[23] Takahashi N, Morimoto S, Okigaki M et al (2011) Decreased plasma level of vitamin C in chronic kidney disease: comparison between diabetic and non-diabetic patients. Nephrol Dial Transplant 26:1252–1257. 10.1093/ndt/gfq547 20817670 10.1093/ndt/gfq547

[24] Luxardo R, Ceretta L, González-Bedat M et al (2022) The Latin American dialysis and renal transplantation registry: report 2019. Clin Kidney J 15:425–431. 10.1093/ckj/sfab188 35211302 10.1093/ckj/sfab188 PMC8862045

[25] Bhatti JS, Sehrawat A, Mishra J et al (2022) Oxidative stress in the pathophysiology of type 2 diabetes and related complications: Current therapeutics strategies and future perspectives. Free Radic Biol Med 184:114–134. 10.1016/j.freeradbiomed.2022.03.019 35398495 10.1016/j.freeradbiomed.2022.03.019

[26] Carr AC, Lykkesfeldt J (2023) Factors affecting the vitamin C dose-concentration relationship: implications for global vitamin C dietary recommendations. Nutrients 15:1657. 10.3390/nu15071657 37049497 10.3390/nu15071657 PMC10096887

[27] Rho MR, Lim JH, Park JH et al (2013) Evaluation of nutrient intake in early post kidney transplant recipients. Clin Nutr Res 2:1. 10.7762/cnr.2013.2.1.1 23429928 10.7762/cnr.2013.2.1.1 PMC3572820

[28] Flannigan KL, Taylor MR, Pereira SK et al (2018) An intact microbiota is required for the gastrointestinal toxicity of the immunosuppressant mycophenolate mofetil. J Heart Lung Transplant 37:1047–1059. 10.1016/j.healun.2018.05.002 30173823 10.1016/j.healun.2018.05.002

[29] Granger DN, Kvietys PR (2015) Reperfusion injury and reactive oxygen species: The evolution of a concept. Redox Biol 6:524–551. 10.1016/j.redox.2015.08.020 26484802 10.1016/j.redox.2015.08.020 PMC4625011

[30] Loong CC, Chang YH, Wu TH, et al (2004) Antioxidant supplementation may improve renal transplant function: A preliminary report. In: Transplantation Proceedings 10.1016/j.transproceed.2004.06.053 15561272

[31] Phillips KM, Tarrago-Trani MT, McGinty RC et al (2018) Seasonal variability of the vitamin C content of fresh fruits and vegetables in a local retail market. J Sci Food Agric 98:4191–4204. 10.1002/jsfa.8941 29406576 10.1002/jsfa.8941

[32] Sotomayor CG, Gomes-Neto AW, Eisenga MF et al (2020) Consumption of fruits and vegetables and cardiovascular mortality in renal transplant recipients: A prospective cohort study. Nephrol Dial Transplant 35:357–365. 10.1093/ndt/gfy248 30165500 10.1093/ndt/gfy248

[33] Collie JTB, Greaves RF, Jones OAH et al (2020) Vitamin C measurement in critical illness: Challenges, methodologies and quality improvements. Clin Chem Lab Med 58:460–470 31829967 10.1515/cclm-2019-0912

[34] Hagel AF, Albrecht H, Dauth W et al (2018) Plasma concentrations of ascorbic acid in a cross section of the German population. J Int Med Res 46:168–174. 10.1177/0300060517714387 28760081 10.1177/0300060517714387 PMC6011295

