# Supplementary material for: Vitamin C deficiency after kidney transplantation: a cohort and cross-sectional study of the TransplantLines biobank
Source: Eur J Nutr. 2024 May 29;63(6):2357–66. doi: 10.1007/s00394-024-03426-7 (PMC11377669; doi:10.1007/s00394-024-03426-7)
Supplement: Supplementary file 1 — Supplementary file1 (DOCX 86 KB) [file 394_2024_3426_MOESM1_ESM.docx]

**Vitamin C deficiency after kidney transplantation: a cohort and cross-sectional study of the TransplantLines biobank**

Manuela Yepes-Calderón ^a^, Yvonne van der Veen ^a^, Fernando Martín del Campo S. ^a,b^, Daan Kremer ^a^, Camilo G. Sotomayor ^a,c,d^, Tim J. Knobbe ^a^, Michel J. Vos ^e^, Eva Corpeleijn ^f^, Martin H. de Borst ^a^, Stephan J. L. Bakker ^a^, TransplantLines Investigators.

^a^ Division of Nephrology, Department of Internal Medicine, University Medical Center Groningen, University of Groningen, Groningen, The Netherlands.

^b^ Faculty of Medicine, Universidad Nacional Autónoma de México, Mexico City, Mexico.

^c^ Clinical Hospital University of Chile, Independencia, Santiago, Chile.

^d^ Institute of Biomedical Sciences, University of Chile, Independencia, Santiago, Chile.

^e^ Department of Laboratory Medicine, University Medical Center Groningen, University of Groningen, Groningen, The Netherlands.^.^

^f^ Department of Epidemiology, University Medical Center Groningen, University of Groningen, Groningen, The Netherlands.

***Supplemental Material***

***SUPPLEMENTAL TABLE OF CONTENT***

**Supplemental Figure 1.** Flow diagram of study population….……………..……………..…3

**Supplemental Figure 2.** Trajectories of vitamin C plasma concentration in KTR….……..…4

**Supplemental Table 1.** Dietary determinants of plasma vitamin C concentration……………5

**Supplemental Table 2.** Food group intake in KTR according to their fish intake…………....6

**Supplemental Table 3.** Effect-modification of baseline characteristics on the associations of Vitamin C intake and supplementation with vitamin C deficiency……………...……………....7


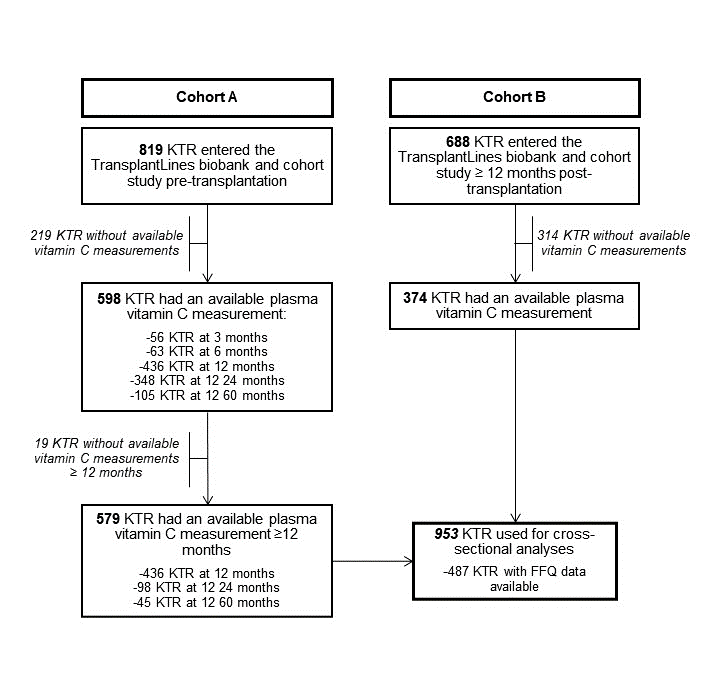
**Supplemental Figure 1. Flow diagram of study population**


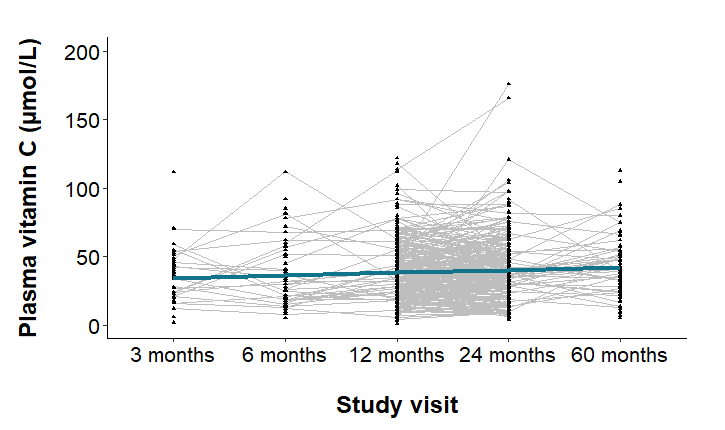


**Supplemental Figure 2. Trajectories of vitamin C plasma concentration in KTR.** Median (IQR) plasma vitamin C concentration was 35 µmol/L (21-48), 30 µmol/L (18-50), 35 µmol/L (22-51), 37 µmol/L (26-52) and 39 µmol/L (26-54) at 3, 6, 12, 24, and 60 months after transplantation, respectively. The blue line represents the average vitamin C trajectory obtained by fitting an unconditional growth model. The average vitamin C change according to this model was +1.57 µmol/L/year.

**Supplemental Table 1.** Dietary determinants of plasma vitamin C concentration

|  |  | **Plasma Vitamin C concentration, µmolL** | | | | |
| --- | --- | --- | --- | --- | --- | --- |
|  |  | **Univariable** | |  | **Adjusted*^†^*** | |
|  |  | **β** | **P value** |  | **β** | **P value** |
| Fruit, 100 g/day |  | 3.25 | <0.001 |  | 2.61 | 0.001 |
| Vegetable, 100 g/day |  | 2.25 | 0.08 |  | 1.71 | 0.17 |
| Dairy, 100 g/day |  | -0.32 | 0.45 |  | 0.01 | 0.98 |
| Meat, 100 g/day |  | 0.78 | 0.57 |  | 0.64 | 0.63 |
| Fish, 100 g/day |  | 8.55 | 0.004 |  | 7.51 | 0.01 |
| Nuts, 100 g/day |  | 11.15 | 0.03 |  | 7.88 | 0.11 |
| Bread, 100 g/day |  | -0.29 | 0.80 |  | -0.50 | 0.65 |
| Koffie, 100 ml/day |  | -0.34 | 0.36 |  | -0.34 | 0.36 |
| Tea, 100 ml/day |  | 0.57 | 0.03 |  | 0.32 | 0.22 |
| Vitamin C intake, 100 mg/day |  | 8.97 | <0.001 |  | 6.30 | 0.002 |
| Vitamin C supplementation use, yes |  | 20.95 | <0.001 |  | 19.94 | <0.001 |

Uni and multivariable linear regression analyses were performed. ***^†^***Adjustment was performed in each logistic regression analysis by diabetes diagnosis, current smoking, HDL cholesterol, eGFR, plasma albumin concentration and time since transplantation.

**Supplemental Table 2.** Food group intake in KTR according to their fish intake.

|  |  | **Tertiles of fish intake^ж^** | | | | |  | **P value^¥^** |
| --- | --- | --- | --- | --- | --- | --- | --- | --- |
|  |  | **Tertile 1** |  | **Tertile 2** |  | **Tertile 3** |  |  |
| Fruit, g/day |  | 143 ± 114 |  | 156 ± 125 |  | 191 ± 126 |  | 0.001 |
| Vegetable, g/day |  | 104 ± 82 |  | 100 ± 66 |  | 140 ± 82 |  | <0.001 |
| Dairy, g/day |  | 334 ± 234 |  | 338 ± 215 |  | 374 ± 266 |  | 0.25 |
| Meat, g/day |  | 108 ± 72 |  | 121 ± 79 |  | 121 ± 70 |  | 0.20 |
| Nuts, g/day |  | 2 (0-9) |  | 4 (1-14) |  | 9 (1-27) |  | <0.001 |
| Bread, g/day |  | 153 ± 97 |  | 170 ± 99 |  | 145 ± 74 |  | 0.03 |
| Koffie, ml/day |  | 420 (120-561) |  | 420 (240-561) |  | 420 (280-561) |  | 0.38 |
| Tea, ml/day |  | 243 (30-680) |  | 243 (18-510) |  | 340 (85-510) |  | 0.21 |

^ж^Tertile 1: <7 g/day, Tertile 2: 7-21 g/day, Tertile3: >21 g/day. ^¥^Differences were tested by ANOVA for continuous variables with normal distribution and Kruskal–Wallis test for continuous variables with non-normal distribution.

**Supplemental Table 3.** Effect-modification of baseline characteristics on the associations of Vitamin C intake and supplementation with vitamin C deficiency.

|  | Vitamin C deficiency | | | |
| --- | --- | --- | --- | --- |
|  | Vitamin C intake | Vitamin C intake^¥^ | Vitamin C supplementation | Vitamin C supplementation^¥^ |
|  | *P for interaction* | *P for interaction* | *P for interaction* | *P for interaction* |
| Age, years | 0.74 | 0.52 | 0.79 | 0.83 |
| Sex, male | 0.66 | 0.69 | 0.14 | 0.33 |
| Diabetes diagnosis, yes | 0.29 | 0.53 | 0.55 | 0.86 |
| Current smoking, yes | 0.69 | 0.49 | 0.09 | 0.18 |
| HDL Cholesterol, mmol/l | 0.69 | 0.60 | 0.28 | 0.54 |
| eGFR, mL/min/1.73 m^2^ | 0.52 | 0.66 | 0.20 | 0.32 |
| Plasma albumin, g/l | 0.38 | 0.18 | 0.92 | 0.87 |
| Time since transplantation, years | 0.49 | 0.54 | 0.50 | 0.42 |

To explore additive interaction, we introduced a multiplicative term between the variable of interest and Vitamin C intake/supplementation in the logistic regression analyses. ^¥^Adjusted for the other variables in the clinical model, plus age and sex.
